# Comparative Study of Different Entry Spots on Postoperative Gluteus Medius Muscle Cross-Sectional Area in Patients With Intertrochanteric Fractures Nailing

**DOI:** 10.7759/cureus.36103

**Published:** 2023-03-13

**Authors:** Mitsuaki Noda, Shunsuke Takahara, Atsuyuki Inui, Keisuke Oe, Shin Osawa, Takehiko Matsushita

**Affiliations:** 1 Department of Orthopaedics, Nishi Hospital, Kobe, JPN; 2 Department of Orthopaedics, Hyogo Prefectural Kakogawa Medical Center, Kakogawa, JPN; 3 Department of Orthopaedic Surgery, Kobe University Graduate School of Medicine, Kobe, JPN; 4 Department of Orthopaedics, Nobuhara Hospital, Tatsuno, JPN

**Keywords:** intertrochanteric femur fracture, comparative study, entry spot, gluteus medius, bald spot, gamma nailing, cross sectional area, cephalo-medullary nailing

## Abstract

Introduction

In a preliminary study of cephalo-medullary (CM) nailing in patients with femoral intertrochanteric fractures, the authors of this study found a 25% to 30% decrease in muscle strength, especially abduction force, during the postoperative follow-up period. This decline was partially attributed to the entry point for the nail insertion causing damage to the gluteus medius tendon at the junction of the greater trochanter after reaming. Therefore, we assumed that changing the position of nail insertion to a "bald spot (BS)" could mitigate postoperative functional impairment. Automated computed tomography (CT) imaging of skeletal muscle cross-sectional area (CSA) and adipose tissue ratio (ATR) can show pathological changes on the operated side compared with the non-operated side. In this study, the authors quantified the difference in postoperative CSA and ATR of the gluteus medius muscle after bald spot nailing versus nail insertion through the conventional tip of the greater trochanter. It was hypothesized that bald spot nailing could avoid significant injury to the gluteus medius muscle.

Materials and methods

Patients with femoral intertrochanteric fractures were grouped according to the site of cephalo-medullary nailing: greater trochanteric tip (TIP) in 27 patients (8 men and 19 women, mean age 84.9±5.1 years) and BS in 16 patients (3 men and 13 women, mean age 86.9±6.2 years). The CSA and ATR of the gluteus medius muscles were assessed in three slices (A, B, and C from proximal to distal). Each slice was manually traced and automatically calculated based on its contour. Adipose tissue (−100 to −50 in Hounsfield units) in the designated area was distinguished by a bimodal image histogram resulting from the distribution of CT numbers of adipose tissue and muscle. The body mass index (BMI) was used to correct the CSA in each patient.

Results

In the TIP group, the mean CSA values (mm^2^) from the non-operated/operated sides were as follows: slice A, 2180.2 ± 616.5/1976.3 ± 421.2; slice B, 2112.3 ± 535.7/1857.7 ± 386.7; and slice C: 1671.8 ± 460.0/1404.1 ± 404.3 (p<0.01 in slices A, B, and C). In the BS group, slice A was 2044.1 ± 473.0/2016.9 ± 388.4; slice B was 2073.2 ± 540.7/1848.3 ± 411.1; and slice C was 1659.1 ± 477.2/1468.5 ± 341.7 (p=0.34 in slice A, and p<0.05 in slices B and C, respectively). The mean CSA values (mm^2^) of the non-operated minus operated side between the TIP/BS groups were as follows: slice A, 241.3 ± 424.3/−11.8 ± 285.6; slice B, 290.3 ± 313.0/211.8 ± 333.2; and slice C, 276.4 ± 270.4/162.8 ± 319.3 (p < 0.05 in slice A, 0.45, 0.24 in slices B, C, respectively). The mean adjusted CSA per BMI values (mm^2^) of the non-operated minus the operated side between the TIP/BS groups were slice A, 10.6 ± 19.7/−0.4 ± 14.8; slice B, 13.3 ± 15.0/10.1 ± 16.3; and slice C, 13.1 ± 13.4/ 8.7 ± 15.3 (p < 0.05 in slice A and 0.54 and 0.36 in slices B and C, respectively).

Conclusion

Nail insertion at the bald spot resulted in a significantly smaller decrease in the CSA of the gluteus medius muscle compared with the conventional tip entry. In addition, an examination of BMI-adjusted CSA showed that CSA was maintained in some image slices. These results suggest that nailing from the BS of the greater trochanter can reduce damage to the gluteus medius muscle and highlight the importance of imaging beyond the usual assessment of skeletal changes.

## Introduction

Cephalo-medullary (CM) nailing is the gold standard treatment for unstable femoral intertrochanteric fractures [1]. During the postoperative period, the gluteus medius muscle stabilizes the pelvis and plays a key role in gait and hip biomechanics [2]. Among the limited number of studies regarding postoperative hip functional score, Cai et al. [3] reported that patients who underwent CM nailing had inferior hip function at 1.5-12 months postoperatively compared with patients who had hip arthroplasty. In our preliminary study, a 25-30% decrease in muscle strength, especially the abduction force, during the postoperative follow-up period was found [4]. In a cadaveric study, McConnell et al. [5] attributed the decline in muscle strength to the entry point for gamma nail insertion, as the damaged area to the gluteus medius tendon at the junction of the greater trochanter after reaming was observed at 27% on average among the cadavers.

Based on such findings, we assumed that changing the position of nail insertion could mitigate postoperative functional impairment. Using an alternative entry point through the piriformis fossa would be advantageous because insertion would follow the femoral axis, resulting in a higher quality of reduction than insertion at the tip of the greater trochanter [6,7]. The use of a slightly medial insertion point was previously reported to cause no injury to the gluteus medius tendon [8]; however, this point of entry may have significant drawbacks. First, it increases the risk of a femoral neck fracture, and second, it threatens the ascending branch of the medial femoral periaortic artery, which could result in femoral necrosis [6]. Another option for the entry point is a bald spot (BS) without tendon insertion that is located lateral to the tip of the greater trochanter. This BS is bordered anteriorly and distally by the gluteus minimus, posteriorly by the gluteus medius, and proximally by the piriformis tendon [9]. It is ideal for femoral nailing because it minimizes the risk of damaging the muscle and prevents future femoral head necrosis [10]. However, studies on the use of BS have been limited to young patients with femoral diaphyseal fractures [11,12].

Automated computed tomography (CT) is widely used to assess gluteal muscle morphology. Assessment of the gluteus muscle, such as its cross-sectional area (CSA), has accelerated since the introduction of the term sarcopenia [13]. In the context of postoperative follow-up, CT imaging of skeletal muscle CSA and adipose tissue ratio (ATR) can show pathological changes on the operated side compared with the non-operated side. The purpose of this current study was to quantify the differences in postoperative CSA and ATR of the gluteus medius muscle in patients with femoral intertrochanteric fractures who underwent BS nailing versus insertion through the conventional tip of the greater trochanter. It was hypothesized that BS nailing might avoid significant injury to the gluteus medius muscle.

## Materials and methods

Patient data sources

**Figure 1:**
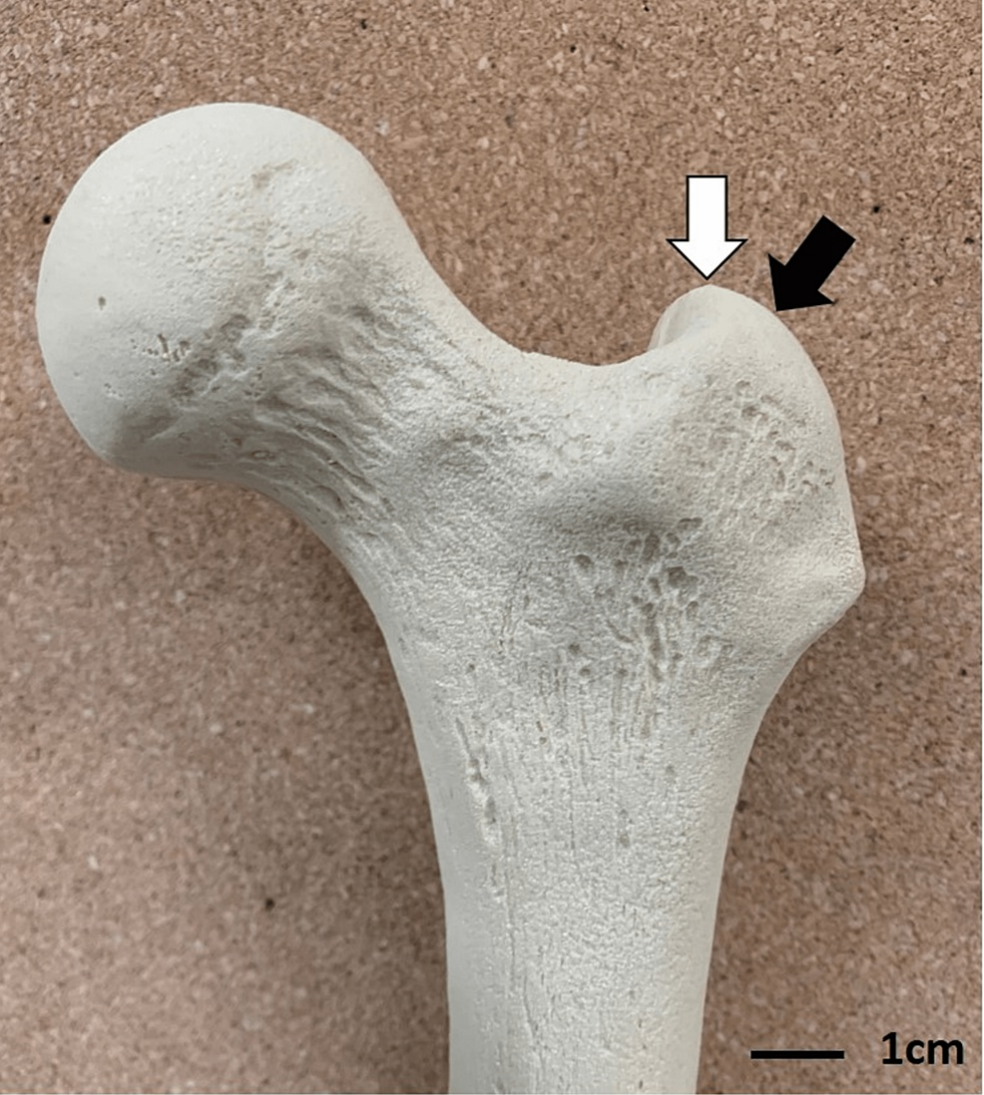
Anatomical spots for nail entry. Greater trochanteric tip in white arrow and bald spot in black arrow.

**Figure 2:**
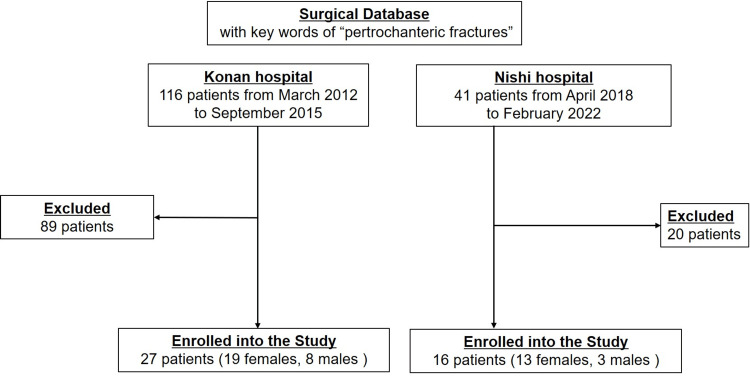
Flowchart of patient selection.

Clinical data of patients with femoral intertrochanteric fractures were retrospectively collected from a trauma database of two hospitals (Konan Hospital and Nishi Hospital, both in Kobe, Japan). Patients with femoral intertrochanteric fractures were classified into the greater trochanteric tip (TIP) and BS groups based on the site of CM nailing (Figure 1). Initially, the TIP group included 116 patients, and the BS group included 47 patients. A CT evaluation was conducted during the postoperative follow-up period. Exclusion criteria were as follows: (1) less than 65 years of age at the time of surgery, (2) less than 10 months of follow-up, (3) bilateral hip surgery, (4) inability to walk, and (5) co-existing central nerve or spinal cord neuropathy, Parkinson’s disease, or other conditions that could affect muscle pathology. After applying the exclusion criteria, we enrolled 27 of the 116 patients from the TIP group and 16 of the 41 patients from the BS group in the study (Figure 2). This study was approved by the ethics committees of both hospitals with verbal and/or written informed consent from each patient (represented by the Nishi Ethics Committee, approval number 2021-1).

Demographic data for the TIP and BS groups

Patient demographic data are presented in Table 1. In the TIP group, eight patients (30%) were male and 19 (70%) were female. The mean age at the time of surgery was 84.9±7.7 years, the mean body weight was 47.8±8.3 kg, and the mean height was 151.1±9.7 cm. Twelve patients were operated on the right side and 15 on the left side. The mean postoperative follow-up for CT scanning was 22 months (range: 11-49 months). In the BS group, three patients were male (19%) and 13 were female (81%). The mean age at the time of surgery was 86.9±6.8 years, the mean body weight was 47.5±7.8 kg, and the mean height was 151.6±7.9 cm. There were eight cases each for right- and left-side operations. The mean postoperative follow-up was 12 months (range: 10-17 months). There was no significant difference between groups, except for the follow-up period.

**Table 1:** Patient demographics and comparison of variables between TIP and BS groups. P-values for two groups were analyzed in each variable using Fisher’s exact probability test for sex, Welch’s t-test for age group, body weight, and height, chi-square test for laterality, and Mann-Whitney U-test for term for CT shooting. ^†^Mean and standard deviation. ^††^Mean and range.

Variable	TIP 27 patients	BS 16 patients	p-value
Sex (number)			
Female	19	13	P=0.34
Male	8	3	
Age group^†^ (years)	84.9±7.7	86.9±6.8	P=0.35
Body weight^†^ (kg)	47.8±8.3	47.5±7.8	P=0.92
Height† (cm)	151.1±9.7	151.6±7.9	P=0.85
Laterality (number)			
Right	12	8	P=0.72
Left	15	8	
Term for CT shooting^††^	22 (11-49)	12 (10-17)	P<0.01

Surgical procedure

**Figure 3:**
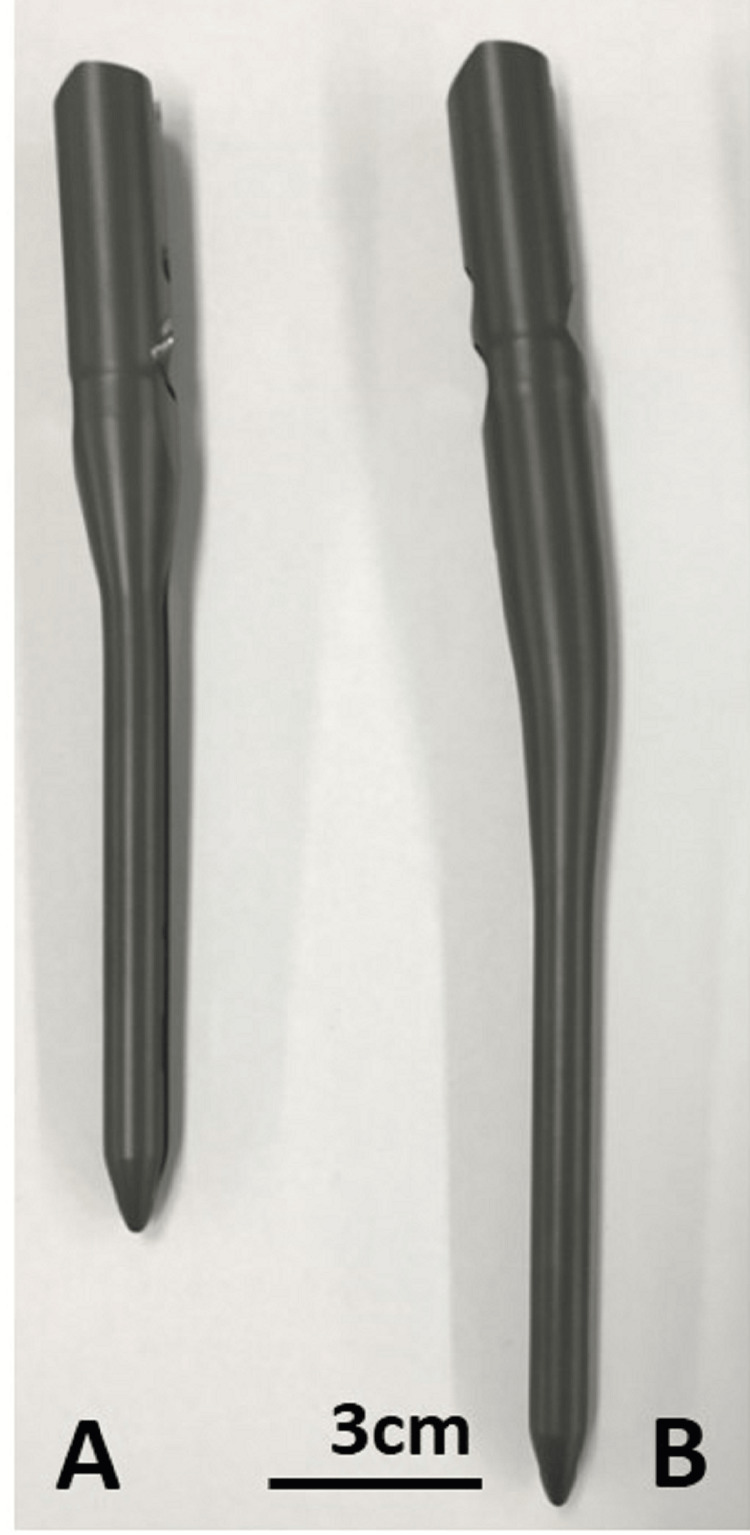
CM nails: (A) conventional nail for TIP entry, curved at 4°; (B) a more curved nail for BS entry at 7°.

CM nailing was performed conventionally. The nails used were Targon PFT (Aesculap AG, Tuttlingen, Germany), with a Targon PFT bending angle of 4° for the TIP group and a Targon PFT Standard curving of 7° for the BS group (Figure 3). In the TIP group, under a two-directional image intensifier, the intramedullary guide pin targeted the greater trochanter and an extension of the proximal femoral shaft, followed by the same 16.5-mm-diameter hole over the guide pin. In the BS group, the same guide pin was displaced outward within 1 cm from the tip of the greater trochanter and was confirmed visually by the surgeon and on the image intensifier. In a considerable number of the patients, an extended skin incision was required distally to anatomically reduce the posteriorly displaced greater trochanter that contained the targeted bald spot. A reduction clamp was applied to maintain this position. A pathway of the same diameter was created with the same crown reamer over the pin. The profiler was manually advanced to enlarge the femoral medullary canal and accommodate the intramedullary nail, followed by the usual surgical procedure. The average length of hospital stay was two to three months, with rehabilitation consisting of a 40-minute daily strength training program and unrestricted early weight-bearing exercise assisted by physical therapists.

CT measurements

**Figure 4:**
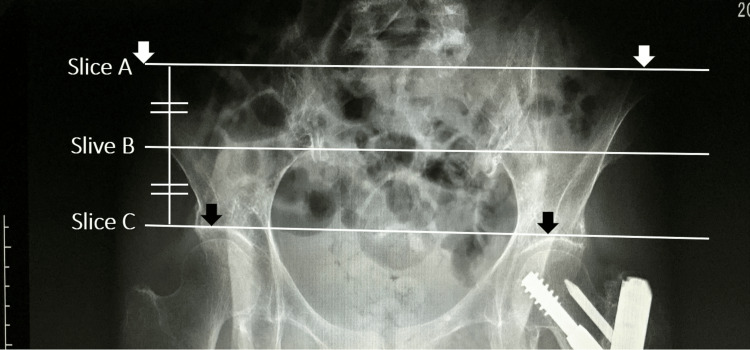
Anatomical location of the three computed tomography scan image slices. Slice A: The line connecting the anterior superior iliac spine (white arrows) bilaterally represents the proximal portion of the gluteus. Slice C: The line connecting both superior margins of the acetabulum (black arrows) indicates the distal portion. Slice B: The midway line between slices A and C.

CT imaging was performed during the postoperative period. A routine scout scan of the pelvis was obtained from each patient, followed by the recording of axial images of the area of the iliac crest to below the lesser trochanter (140 kVp, auto mA, noise index 13; and 120 kVp, auto mA, noise index 9.5). Slices were 10.0 mm thick at 10.0 mm intervals and 5.0 mm thick at 55.0 mm intervals, respectively. A Phillips MX 8000 (Phillips Medical Systems, Cleveland, OH, USA) spiral CT scanner was used in the TIP group, and the Optima CT 660 CT Scan (GE Healthcare Japan, Tokyo, Japan) was used in the BS group. Scan images were acquired using the Advantage Work Station (Version 4.6; GE Healthcare Japan) and AZE Virtual Place Fujin Raijin (Version 3.0, AZE, Tokyo, Japan), respectively. Images were converted to digital imaging and communications in medicine (DICOM) format (512 × 512 pixels) and modified to quantify the CSA of muscle, bone, and fat to the nearest 0.01 cm2. Prior to CSA measurements, the examiner carefully acquired three slices parallel to bilaterally symmetrical anatomical landmarks: (1) slice A, view connecting the bilateral superior iliac spines; (2) slice C, line connecting both superior margins of the acetabulum; and (3) slice B, midway between slices A and C (Figure 4). The CSA and ATR of the gluteus medius muscles were assessed in slices A, B, and C. Radiographic technicians were instructed to correct pelvic rotation to maintain a symmetrical view.

**Figure 5:**
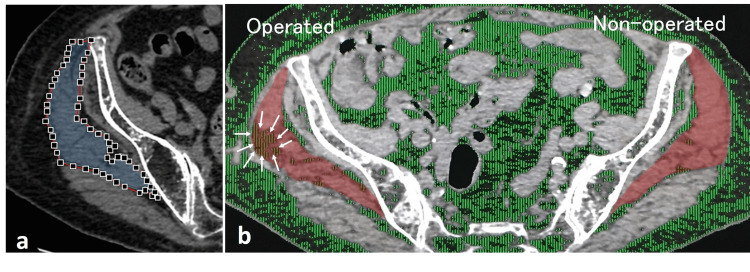
CSA of the gluteus medius muscle. (a) Observers manually dotted boundaries of the muscle to scale the area. (b) Gluteus medius in red in the TIP group. The CSA on the operated side is smaller than the contralateral non-operated side. There is obvious adipose tissue proliferation (white arrows) on the operated side, reflecting the path of nail insertion.

The CSA was manually traced and automatically calculated based on its contour (Figure 5). Adipose tissue (−100 to −50 in Hounsfield units) in the designated area was distinguished by a bimodal image histogram resulting from the distribution of CT numbers of adipose tissue and muscle [14]. The ATR was expressed bilaterally as the ratio of the area of intramuscular adipose tissue to the CSA [15]. The CSA was measured three times on both the non-operated and operated sides by three independent examiners in the TIP group. The averages of the three measurements were calculated, and the average values on the operated and non-operated sides were compared. CSA was only measured once by a single examiner in the BS group.

Body mass index (BMI, kg/m^2^) was used to correct the muscle area for each patient, representing a CSA per person [16].

Statistical analysis

All statistical analyses were performed using EZR (Saitama Medical University Hospital, Saitama, Japan), a graphical user interface of R (The R Foundation for Statistical Computing, Vienna, Austria). Statistical significance was set at p < 0.05. For the demographic data, chi-square or Fisher’s exact probability tests were used to assess the significance of each parameter of the categorical scale described as counts between two groups. The Kolmogorov-Smirnov test was used to determine the normality of continuous variables and is presented as mean ± standard deviation. Welch’s t-test was used for numerical variables with normality. Non-normally distributed data were analyzed using the Mann-Whitney U-test.

The CSA and ATR values on the operated and non-operated sides in each group were compared. A paired t-test was used for parametric data, whereas the Wilcoxon signed rank test was substituted for non-parametric groups to test differences.

The effect of changing the position of nail insertion on CSA and ATR was examined between the TIP and BS groups. Since the diameter of the nail was the same, muscle damage for each patient was assessed by subtracting the value of the operated side from the value of the non-operated side for each group. Welch’s t-test was used for parametric data, and the Mann-Whitney U-test was used for non-parametrically distributed data. The same comparison, subtracting the value of bilateral sides, was also made for adjusted CSA between two groups per BMI (CSA/weight, mm^2^/kg).

## Results

CSA measurement

In the TIP group, the mean CSA values (mm^2^) from the non-operated/operated sides were as follows: slice A, 2180.2 ± 616.5/1976.3 ± 421.2; slice B, 2112.3 ± 535.7/1857.7 ± 386.7; and slice C, 1671.8 ± 460.0/1404.1 ± 404.3 (Table 2). The CSA values measured from all slices were significantly lower on the operated side compared with the non-operated side (p=0.003, 0.001, and 0.001 in slices A, B, and C, respectively). In the BS group, the mean CSA values (mm^2^) from the non-operated/operated sides were as follows: slice A, 2044.1 ± 473.0/2016.9 ± 388.4; slice B, 2073.2 ± 540.7/1848.3 ± 411.1; and slice C, 1659.1 ± 477.2/1468.5 ± 341.7. The CSA values were similar between the non-operated and operated sides in slice A, but were significantly different in slices B and C (p=0.34, 0.03, and 0.04 in slices A, B, and C, respectively).

**Table 2:** Values of cross-sectional area of the gluteus medius muscle at slices A to C, in the operated side and non-operated contralateral side. The values in TIP are given as the mean and the standard deviation. Twenty-seven CSAs of gluteus medius were analyzed. The values in BS are given as the mean and the standard deviation. Sixteen CSAs of gluteus medius were analyzed. *Significance.

	Non-operated side	Operated side	P-value
TIP			
Slice A	2180.2±616.5	1976.3±421.2	0.003*
Slice B	2112.3±535.7	1857.7±386.7	0.001*
Slice C	1671.8±460.0	1404.1±404.3	0.001*
BS			
Slice A	2044.1±473.0	2016.9±388.4	0.34
Slice B	2073.2±540.7	1848.3±411.1	0.03*
Slice C	1659.1±477.2	1468.5±341.7	0.04*

ATR measurement

In the TIP group, the mean ATR values (%) from the non-operated/operated sides were as follows: slice A, 2.8 ± 1.7/5.2 ± 3.5; slice B, 2.7 ± 1.9/4.6 ± 3.2; and slice C, 3.6 ± 3.0/4.8 ± 3.2 (Table 3). The ATR in the TIP of the gluteus medius muscle in all slices was significantly higher on the operated side compared with the non-operated side (p < 0.01 in slices A and B and p = 0.99 in slice C). In the BS group, the mean ATR values (%) from the non-operated/operated sides were as follows: slice A, 5.4 ± 3.9/8.6 ± 4.2; slice B, 3.8 ± 2.5/8.2 ± 5.8; and slice C, 5.1 ± 3.1/8.8 ± 6.5. The ATR in the CSA of the gluteus medius muscle was significantly different only in slice C (p=0.99, 0.99, and 0.004 in slices A, B, and C, respectively).

**Table 3:** Distribution of adipose tissue ratio in the gluteus medius muscle at slices A to C, in the operated side and non-operated contralateral side. The values in TIP are given as the mean and the standard deviation. Twenty-seven ATRs of gluteus medius were analyzed. The values in BS are given as the mean and the standard deviation. Sixteen ATRs of gluteus medius were analyzed. *Significance.

	Non-operated side	Operated side	P-value
TIP (%)			
Slice A	2.8±1.7	5.2±3.5	0.01*
Slice B	2.7±1.9	4.6±3.2	0.01*
Slice C	3.6±3.0	4.8±3.2	0.99
BS (%)			
Slice A	5.4±3.9	8.6±4.2	0.99
Slice B	3.8±2.5	8.2±5.8	0.99
Slice C	5.1±3.1	8.8±6.5	0.004*

Comparison of CSA, ATR, and adjusted CSA between TIP and BS groups

The mean CSA values (mm^2^) of the non-operated minus operated side between the TIP/BS groups were as follows: slice A, 241.3 ± 424.3/−11.8 ± 285.6; slice B, 290.3 ± 313.0/211.8 ± 333.2; and slice C, 276.4 ± 270.4/162.8 ± 319.3 (Table 4). A significant difference was observed only for slice A (p=0.02, 0.45, and 0.24 in slices A, B, and C, respectively). The mean ATR values (%) of the non-operated minus the operated side between the TIP/BS groups were the following: slice A, −2.4 ± 2.7/−3.3 ± 2.5; slice B, −1.9 ± 2.4/−4.3 ± 4.0; and slice C, −1.3 ± 2.3/−3.6 ± 5.2 showing significant difference in slice B (p=0.10, 0.04, 0.12 in slices A, B, and C, respectively) (Table 5). The mean adjusted CSA per BMI values (mm^2^) of the non-operated minus the operated side between the TIP/BS groups were the following: slice A, 10.6 ± 19.7/−0.4 ± 14.8; slice B, 13.3 ± 15.0/10.1 ± 16.3; and slice C, 13.1 ± 13.4/8.7 ± 15.3 (Table 6). A significant difference was observed only in slice A (p=0.049, 0.54, and 0.36 in slices A, B, and C, respectively).

**Table 4:** Comparison of values of cross-sectional area between trochanteric tip and bald spot groups. The difference of values (non-operative side minus operative side) is given as the mean and the standard deviation. CSA of gluteus medius was analyzed. *Significance.

	TIP (non-ope) –ope	BS (non-ope) –ope	P-value
Slice A	241.3±424.3	−11.8±285.6	0.02*
Slice B	290.3±313.0	211.8±333.2	0.45
Slice C	276.4±270.4	162.8±319.3	0.24

**Table 5:** Comparison of values of adipose tissue ratio between trochanteric tip and bald spot groups. The difference of values (non-operative side minus operative side) are given as the mean and the standard deviation. *Significance.

-	TIP (non-ope) –ope	BS (non-ope) –ope	P-value
Slice A	−2.4±2.7	−3.3±2.5	0.10
Slice B	−1.9±2.4	−4.3±4.0	0.04*
Slice C	−1.3±2.3	−3.6±5.2	0.12

**Table 6:** Comparison of values of body mass index-adjusted cross-sectional area between trochanteric tip and bald spot groups. The difference of values (non-operated side minus operated side) are given as the mean and the standard deviation. *Significance.

	TIP (non-ope) –ope	BS (non-ope) –ope	P-value
Slice A	10.6±19.7	−0.4±14.8	0.049*
Slice B	13.3±15.0	10.1±16.3	0.54
Slice C	13.1±13.4	8.7±15.3	0.36

## Discussion

The current study demonstrated that with the use of the BS for insertion, the decrease in mean CSA values measured on the operated side was from 80 to 200 mm2 in comparison with the contralateral non-operated side. In contrast, the mean CSA values with conventional nailing were reduced by approximately 200 mm2 or less than 300 mm2 on the operated side compared with the non-operated side. The comparison of the CSA and adjusted CSA values of the non-operated side minus the operated side between the TIP and BS groups demonstrated a significant difference in slice A, while the ATR values were significantly different in slice B. These results suggest that nailing from the BS of the greater trochanter may reduce damage to the gluteus medius muscle, which is consistent with our hypothesis.

Surgical technique preparation

Even though this method does not have major technical changes, a surgeon adopting it should be aware of several important points. First, instead of the traditional 4°-bent nail, a nail with more curvature should be prepared, especially to accommodate the shorter skeletal structure of the Asian population. In a study of 98 elderly patients with intertrochanteric fractures, Pan et al. [10] reported that a laterally located entry spot with a normal implant helical blade resulted in five iatrogenic lateral proximal fractures, and about 30% of head screws were placed in a non-recommended upward position. Second, not all patients are eligible for the 7° curved implant. In our experience, the bended profiler for nail insertion cannot be used in about 20% of patients. In such cases, surgeons should consider a conventional profiler and insert a conventional 4°-bent implant. Third, a displaced greater trochanter needs to be anatomically aligned and repositioned because the BS is often located in this fragment. This procedure requires an extension of the skin incision and open reduction, as the external rotators contract it posteriorly.

It should be noted that insertion from the BS decreases injury to the gluteus medius muscle to some extent, but it does not necessarily lead to a complete absence of muscle damage for two reasons. First, the nail hole at the BS may cause partial muscular damage. Gardner et al. [9] revealed a bony footprint of 20-mm-diameter free space; however, the distance between this posterior facet and the medial facet was narrower in a Japanese cadaveric study, averaging approximately 15 mm [17]. A 16.5-mm reamer would make a hole that could cause some muscle damage. Second, the gluteus medius tendon is assumed to be compromised. It has been anatomically proven that the BS has tendons running across its surface, even if direct muscle attachment is absent [17]. Therefore, although entry through the BS may reduce direct muscular damage, indirect tendon compromise is inevitable.

Our clinical experience supports the results of this study. First, as an intraoperative finding, less volume of muscle was extracted in a cylindrical crown reaming for BS. This finding was in accordance with a study of 34 cadavers, showing a 27% damage in the average CSA of the gluteus medius muscle in 27% of cases [5]. Second, the BS group showed less abductor muscle weakness than the TIP group in manual muscle testing at approximately one year follow-up, and these clinical outcomes support the present results.

Even though CT imaging has been used to measure muscle size and quantify muscle pathology associated with hip fractures, few studies have compared the fractured and contralateral sides [15]. A number of studies using CT imaging of the gluteus muscle and the waist and thigh show the relationship between sarcopenia and life expectancy [13,18-21]. For example, a lower psoas CSA measured by a CT scan has been associated with a loss of independence upon discharge from the hospital [20]. In addition, changes in muscle density have been reported to precede hip fracture and to be a long-term predictor of frailty leading to hip fracture [19,20]. Jung et al. [13] studied the preoperative and postoperative changes of the CSA and attenuation around the hip joint only on the non-operated side to examine preoperative and postoperative changes. The current paper focuses on bilateral CSA as one of the indicators for predicting postoperative function. To date, there is no English-language literature analyzing the effect of nail placement by comparing the CSA or ATR of the gluteus medius muscle.

Strengths

This study has several strengths. First, all patients remained hospitalized in the same facilities for at least two months while undergoing adequate rehabilitation directed by the attending physician, who was familiar with the intraoperative findings and the patient’s overall condition. Second, the follow-up rate exceeded 90% [21]. In contrast, Aspenberg et al. [22] reported a very high dropout rate and a low compliance visit rate among aged patients in a six-month follow-up period. The high follow-up rate in our study is due to the fact that most patients resided in affiliated nursing homes or in neighboring communities that had easy access to our institutions.

Limitations

Similarly, several limitations of this study must also be acknowledged. First, it was not possible to equalize the number of patients in each group or the modality of CT imaging due to the different systems between the two hospitals. Second, approximately three-quarters of the patients in the database were excluded, leading to smaller sample sizes for both groups. Most of the elderly patients were fragile and hesitant to walk for personal safety reasons. Therefore, only relatively healthy ambulatory patients were recruited for this study. Third, regarding the BS data, only one examiner measured CSA and ATR, compared with three surgeons for the TIP group. Fourth, the follow-up period of the BS group was only 12 months on average. A longer follow-up period is ideal, as some reports emphasize two years for complete muscle recovery [23,24]. However, the difficulty of sustaining a longer follow-up period in older patients must be taken into account.

Future direction

Studies of intertrochanteric fractures have often been based on simple radiographic imaging focused on bone union or displacement and simple functional questionnaires [25]. However, in this present study, it is also believed to be important to assess soft tissue impairment using CT images or other modern imaging apparatus. In addition, it is necessary to investigate the relationship between decreased CSA and decreased abduction strength or functional impairment.

## Conclusions

The current study demonstrated that the use of the BS for insertion led to the CSA values of the gluteus medius muscle in the non-operated side and the operated side being almost similar in one slice (no statistically significant difference), whereas with the use of the conventional insertion, the CSA values of the operated side were significantly decreased in comparison with the contralateral non-operated side in all slices. Nail insertion at the BS resulted in a significantly smaller decrease in the CSA compared with the conventional tip entry. In addition, an examination of BMI-adjusted CSA showed that CSA was maintained in some image slices.

These results suggest that nailing from the BS of the greater trochanter can reduce damage to the gluteus medius muscle, although some muscle damage is inevitable. These studies shed light on the importance of imaging beyond the usual assessment of skeletal changes.

.
